# The inverse scattering problem for orthotropic media in polarization-sensitive optical coherence tomography

**DOI:** 10.1007/s13137-017-0102-y

**Published:** 2017-12-27

**Authors:** Peter Elbau, Leonidas Mindrinos, Otmar Scherzer

**Affiliations:** 1 Computational Science Center, University of Vienna, Oskar-Morgenstern-Platz 1, 1090 Vienna, Austria; 2 Johann Radon Institute for Computational and Applied Mathematics (RICAM), Altenbergerstraße 69, 4040 Linz, Austria

**Keywords:** Optical coherence tomography, Maxwell’s equations, Electromagnetic scattering, Nonlinear integral equation, 35R30, 35Q61, 45B05, 45G15, 78A46

## Abstract

In this paper we provide for a first time, to our knowledge, a mathematical model for imaging an anisotropic, orthotropic medium with polarization-sensitive optical coherence tomography. The imaging problem is formulated as an inverse scattering problem in three dimensions for reconstructing the electrical susceptibility of the medium using Maxwell’s equations. Our reconstruction method is based on the second-order Born-approximation of the electric field.

## Introduction

Optical coherence tomography (OCT) is an imaging technique producing high-resolution images of the inner structure of biological tissues. Standard OCT uses broadband, continuous wave light for illumination and the images are obtained by measuring the time delay and the intensity of the backscattered light from the sample. For a detailed description of OCT systems we refer to the books (Bouma and Tearney 2002; Drexler and Fujimoto 2015) and for a mathematical modeling we refer to Elbau et al. (2015).

Apart form standard OCT, there exist also functional OCT techniques such as the polarization-sensitive OCT (PS-OCT) which considers the differences in the polarization state of light to determine the optical properties of the sample. PS-OCT is based on polarization-sensitive low coherence interferometry established by Hee et al. (1992) and then first applied to produce two-dimensional OCT images (de Boer et al. 1997, 1998). In this work, we consider the basic scheme of a PS-OCT system which consists of a Michelson interferometer with the addition of polarizers and quarter-wave plates (QWP).

**Fig. 1:**
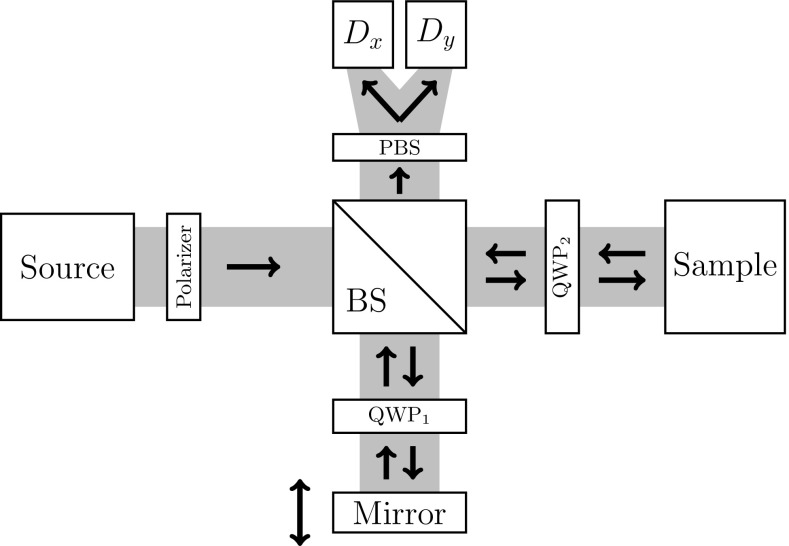
Schematic representation of the light travelling in a time-domain PS-OCT system. In the reference and sample arms are placed quarter-wave plates (QWP) at specific orientations

More precisely, a linear polarizer is added after the source and the linear (horizontal or vertical) polarized light is split into two identical parts by a polarization-insensitive beam splitter (BS). In the reference arm the light is reflected by a mirror and in the sample arm the light is incident on the medium. At the BS, the back-reflected beam and the backscattered light from the sample, in an arbitrary polarization state, are recombined. The recombined light passes through a polarizing BS which splits the output signal into its horizontal and vertical components to be measured at two different photo detectors. See Fig. 1 for an illustration of this setup.

To describe the change in the polarization state of the light due to its propagation into the sample we adopt the analysis based on the theory of electromagnetic waves scattered by anisotropic inhomogeneous media (Colton and Kress 2013; Wolf and Foley 1989). We assume that the dielectric medium is linear and anisotropic. In addition, we impose the property that the medium is invariant under reflection by the $x_1{-}x_2$ plane. A medium with this property is called orthotropic in the mathematical community (Cakoni and Colton 2014) or monoclinic in the material science community (Torquato 2002).

The medium is also considered as weakly scattering and we present the solution in the accuracy of the second-order Born-approximation. As we are going to see later, we consider higher-order approximation in order to be able to recover all the material parameters. We describe the change in the polarization state of the light by the Jones matrix formalism which is applicable since OCT detects the coherent part of the electric field of the backscattered light (Jiao and Wang 2002). As in standard OCT systems, the backscattered light is detected in the far field.

In the medical community, the sample is usually described by a general retarder and the change in the polarization state of the light returning from the sample can be modelled by a Jones matrix (Hitzenberger et al. 2001; Jiao and Wang 2002). However, even though the produced images are satisfactory they are mainly used qualitatively. The usage of these images comes only secondarily to quantify the optical parameters by image processing techniques.

In this work we are interested in the quantitative description of PS-OCT. To do so, we have first to describe mathematically the system properly. Thus, we represent the polarized scattered field as solution to the full-wave Maxwell’s equations. This has not yet been applied to PS-OCT, since for isotropic media, the Born-approximation decouples the effects of the optical properties of the sample from the polarization state of the scattered field. However, this analysis for anisotropic media provides enough information to reconstruct the electric susceptibility of the medium. The scattered field satisfies then an integral equation of Lippmann–Schwinger type. Under the far-field approximation and the assumption of a homogeneous background medium we obtain a system of integral equations for the unknown optical parameters.

In the mathematical literature, the scattering problem by anisotropic objects has been widely considered over the last decades (Beker and Umashankar 1989; Geng et al. 2003; Graglia and Uslenghi 1984; Papadakis et al. 1990). Recently, the connection between the inverse problem to reconstruct the refractive index and the interior transmission problem has been investigated (Cakoni et al. 2010; Cakoni and Haddar 2007). For the specific case of an orthotropic medium we refer the reader to the book (Cakoni and Colton 2014) and to Colton et al. (1997) and Potthast (1999) for results concerning the uniqueness and existence of solutions of the inverse problem.

The paper is organized as follows: In Sect. 2, we derive the integral representation of the scattered field for an orthotropic medium in the accuracy of the second-order Born-approximation in the far-field zone. In Sect. 3, we describe mathematically the standard PS-OCT system using the Jones matrix formalism and we derive the expression for the cross-spectral density. The system of equations for all the components of the susceptibility is presented in the last section using two incident illuminations.

### Notation

In this paper, we use the following conventions:

- Let $f\,{:}\,\mathbb {R}\rightarrow \mathbb {C}$ be integrable, then the one-dimensional Fourier-transform is defined by
  $$\begin{aligned} \hat{f}(\omega ) = \int _{\mathbb {R}} f(t)\mathrm{e}^{\mathrm{i}\omega t}{\,\mathrm d}t, \quad \text{ for } \text{ all }\quad \omega \in \mathbb {R}. \end{aligned}$$
- Let $f\,{:}\,\mathbb {R}\rightarrow \mathbb {C}$ be integrable, then the one-dimensional inverse Fourier-transform is defined by
  $$\begin{aligned} \check{f}(t) = \frac{1}{2\pi }\int _{\mathbb {R}} f(\omega )\mathrm{e}^{-\mathrm{i}\omega t}{\,\mathrm d}\omega , \quad \text{ for } \text{ all }\quad t \in \mathbb {R}. \end{aligned}$$
- Let $f\,{:}\,\mathbb {R}^3 \rightarrow \mathbb {C}$ be integrable, then the three-dimensional Fourier-transform is defined by
  $$\begin{aligned} \tilde{f}(\varvec{k}) = \int _{\mathbb {R}^3} f(\varvec{x})\mathrm{e}^{-\mathrm{i}\left<\varvec{k},\varvec{x}\right>} {\,\mathrm d}\varvec{x}, \quad \text{ for } \text{ all }\quad \varvec{x} \in \mathbb {R}^3. \end{aligned}$$

## The direct scattering problem

In absence of external charges and currents, the propagation of electromagnetic waves in a non-magnetic medium is mathematically described by Maxwell’s equations relating the electric and magnetic fields ${\varvec{E}}\,{:}\,\mathbb {R}\times \mathbb {R}^3 \rightarrow \mathbb {R}^3$ and $\varvec{H}\,{:}\,\mathbb {R}\times \mathbb {R}^3 \rightarrow \mathbb {R}^3$ and the electric displacement $\varvec{D}\,{:}\,\mathbb {R}\times \mathbb {R}^3 \rightarrow \mathbb {R}^3$ by1where *c* is the speed of light. Maxwell’s equations are not sufficient to uniquely determine the fields $\varvec{D}, {\varvec{E}}$ and $\varvec{H}$. Therefore additional material parameters have to be specified:

### Definition 1

An anisotropic medium is called *linear dielectric* if there exists a function, called the *electric susceptibility*,

$$\begin{aligned} {\varvec{\chi }}\in C^\infty _{\mathrm c}(\mathbb {R}\times \mathbb {R}^3; \mathbb {R}^{3\times 3}),\quad \text {with}\quad {\varvec{\chi }}(\tau ,\varvec{x})=0\quad \text { for all }\quad \tau <0, \varvec{x}\in \mathbb {R}^3, \end{aligned}$$

satisfying

2 $$\begin{aligned} \varvec{D} (t,\varvec{x}) = {\varvec{E}}(t, \varvec{x}) + \int _{\mathbb {R}} {\varvec{\chi }}(\tau ,\varvec{x}){\varvec{E}}(t-\tau ,\varvec{x}){\,\mathrm d}\tau . \end{aligned}$$

A linear dielectric medium is called *orthotropic* (Cakoni and Colton 2014; Colton et al. 1997) if it admits the special symmetric form

3 $$\begin{aligned} {\varvec{\chi }}= \begin{pmatrix} \chi _{11} &{}\quad \chi _{12} &{}\quad 0 \\ \chi _{12} &{}\quad \chi _{22} &{}\quad 0 \\ 0 &{}\quad 0 &{}\quad \chi _{33} \end{pmatrix}. \end{aligned}$$

Application of the Fourier transform to Maxwell’s equations (1) and taking into account (2), it follows that the Fourier-transform $\widehat{\varvec{E}}$ of ${\varvec{E}}$ satisfies the *vector Helmholtz equation*

4 $$\begin{aligned} \nabla \times \nabla \times \widehat{\varvec{E}}(\omega ,\varvec{x}) - \frac{\omega ^2}{c^2}({\mathbb {1}}+\hat{{\varvec{\chi }}}(\omega ,\varvec{x})) \widehat{\varvec{E}}(\omega , \varvec{x}) = 0, \quad \omega \in \mathbb {R},\quad \varvec{x}\in \mathbb {R}^3. \end{aligned}$$

### Definition 2

We call an electric field ${\varvec{E}}^{i}\,{:}\,\mathbb {R}\times \mathbb {R}^3\rightarrow \mathbb {R}^3$ a *causal initial field* (CIF) with respect to some domain $\varOmega \subseteq \mathbb {R}^3$ if

1. Its Fourier transform with respect to time solves Maxwell’s equations (1) with a susceptibility ${\varvec{\chi }}= 0$, that is,
  5 $$\begin{aligned} \nabla \times \nabla \times \widehat{\varvec{E}}^{i}(\omega ,\varvec{x})- \frac{\omega ^2}{c^2} \widehat{\varvec{E}}^{i}(\omega ,\varvec{x})= 0,\quad \text {and}\quad \nabla \cdot \widehat{\varvec{E}}^{i}(\omega ,\varvec{x}) = 0,\quad \omega \in \mathbb {R},\;\varvec{x}\in \mathbb {R}^3, \end{aligned}$$
2. and satisfies $$\text{ supp } {\varvec{E}}^{i}(t,\cdot ) \cap \varOmega = \emptyset \text { for every } t \le 0$$.

The second condition means that ${\varvec{E}}^{i}$ does not interact with the medium contained in $\varOmega$ until the time $t=0.$

### Example 1

Let $\varOmega \subset \mathbb {R}^3$ be an open set, such that $\text{ supp } {\varvec{\chi }}(t,\cdot ) \subset \varOmega$ for all $t \in \mathbb {R}.$ Moreover, let $\varvec{q}\in \mathbb {R}^2\times \{0\}$ (denoting the polarization vector), $f \in C^\infty _{\mathrm c}(\mathbb {R})$ and

6 $$\begin{aligned} {\varvec{E}}^{0} (t,\varvec{x})= \varvec{q} f\left( t+\tfrac{x_3}{c}\right) , \end{aligned}$$

such that

$$\begin{aligned} \text{ supp } {\varvec{E}}^{0} (t,\cdot ) \cap \varOmega = \emptyset \quad \text {for every}\quad t \le 0. \end{aligned}$$

Then ${\varvec{E}}^{0}$ is a CIF.

### Proof

To see this note that for arbitrary $\varvec{q} \in \mathbb {R}^3$ we get

$$\begin{aligned} \nabla \times \nabla \times {\varvec{E}}^{0}&= \nabla \times \left( \frac{1}{c} f'\left( t+\tfrac{x_3}{c}\right) \,\varvec{e}_3 \times \varvec{q}\right) = \frac{1}{c^2} f''\left( t+\tfrac{x_3}{c}\right) \,\varvec{e}_3 \times (\varvec{e}_3 \times \varvec{q} ) \\&= -\frac{1}{c^2} f''\left( t+\tfrac{x_3}{c}\right) \varvec{q} =-\frac{1}{c^2} \partial _{tt}{\varvec{E}}^{0}. \end{aligned}$$

And for the particular choice $\varvec{q}\in \mathbb {R}^2\times \{0\}$ we even have that $\nabla \cdot {\varvec{E}}^{0}=0$. This shows that ${\varvec{E}}^{0}$ is a solution of Maxwell’s equation. The second assertion is an immediate consequence of the second assumption. $\square$

### Theorem 1

Let ${\varvec{E}}^{i}$ be a CIF-function and assume that the susceptibility ${\varvec{\chi }}$ represents a dielectric, orthotropic medium. Then,

1. there exists a solution ${\varvec{E}}$ (together with $\varvec{H}$) of Maxwell’s equations (1) which satisfies
  7 $$\begin{aligned} {\varvec{E}}(t,\varvec{x}) = {\varvec{E}}^{i}(t,\varvec{x}),\quad t \le 0,\quad \varvec{x}\in \mathbb {R}^3. \end{aligned}$$
2. For every $\varvec{x} \in \mathbb {R}^3$ the function
  $$\begin{aligned} \begin{aligned} g: \mathbb {R}&\rightarrow \mathbb {C}\,,\\ \omega&\mapsto (\widehat{\varvec{E}}- \widehat{\varvec{E}}^{i})(\omega ,\varvec{x}), \end{aligned} \end{aligned}$$
  can be extended to a square integrable, holomorphic function on the upper half plane
  $$\begin{aligned} \mathbb {H}=\{ \omega \in \mathbb {C}\mid \mathfrak {I}{(\omega )} >0 \}. \end{aligned}$$
3. $\widehat{\varvec{E}}$ solves the *Lippmann–Schwinger integral equation*
  8 $$\begin{aligned} \begin{aligned} \widehat{\varvec{E}}(\omega ,\varvec{x})&= \widehat{\varvec{E}}^{i}(\omega ,\varvec{x})+\left( \frac{\omega ^2}{c^2} \mathbb {1}+\nabla \nabla \cdot \right) \int _{\mathbb {R}^3}G(\omega , \varvec{x}-\varvec{y})\hat{{\varvec{\chi }}}(\omega ,\varvec{y})\widehat{\varvec{E}}(\omega ,\varvec{y}){\,\mathrm d}\varvec{y}\\&=: \widehat{\varvec{E}}^{i}(\omega ,\varvec{x}) + \varvec{\mathcal {G}}[\hat{{\varvec{\chi }}} \widehat{\varvec{E}}](\omega ,\varvec{x})\,, \end{aligned} \end{aligned}$$
  where
  $$\begin{aligned} G(\omega , \varvec{x}) = \frac{\mathrm{e}^{\mathrm{i}\frac{\omega }{c}| \varvec{x}|}}{4\pi |\varvec{x}|}, \quad \varvec{x} \ne 0, \quad \omega \in \mathbb {R}\end{aligned}$$
  is the fundamental solution of the scalar Helmholtz equation.

The integral operator $\varvec{\mathcal {G}}$ is strongly singular and we address its properties in the last section.

### Proof

From the initial condition (7) it follows for every solution ${\varvec{E}}$ of Maxwell’s equations (1) which fulfills (7) that the inverse Fourier-transform of *g* satisfies

$$\begin{aligned} \check{g}(t)=0\quad \text {for all}\quad t \le 0. \end{aligned}$$

Thus, the second assertion is a direct consequence of the Paley–Wiener theorem (Papoulis 1962).

To prove the first part, note that the electric field $\widehat{\varvec{E}}$ is uniquely defined by (4) together with the assumption that the function *g* can be for every $x\in \mathbb {R}^3$ extended to a square integrable, holomorphic function on the upper half plane.

Finally, the solution of Eq. (4) can be written as the solution of the integral Eq. (8), see Cakoni and Colton (2014) and Potthast (2000).$\square$

### Born and far-field approximation

We assume that the medium is weakly scattering, meaning that $\hat{{\varvec{\chi }}}$ is sufficiently small (Chew 1990; Colton and Kress 2013) such that the incident field $\widehat{\varvec{E}}^{i}$ is significantly larger than $\widehat{\varvec{E}}-\widehat{\varvec{E}}^{i}$.

#### Definition 3

The *first order* Born-approximation of the solution $\widehat{\varvec{E}}$ of the Lippmann–Schwinger equation (8) is defined by

9 $$\begin{aligned} \widehat{\varvec{E}}^{1} = \widehat{\varvec{E}}^{i}+ \varvec{\mathcal {G}}[\hat{{\varvec{\chi }}} \widehat{\varvec{E}}^{i}]. \end{aligned}$$

The *second order* Born-approximation is defined by

10 $$\begin{aligned} \widehat{\varvec{E}}^{2} = \widehat{\varvec{E}}^{i}+ \varvec{\mathcal {G}}[\hat{{\varvec{\chi }}} \widehat{\varvec{E}}^{1} ]. \end{aligned}$$

Inserting (9) into (10) gives

11 $$\begin{aligned} \widehat{\varvec{E}}^{2} = \widehat{\varvec{E}}^{i}+ \varvec{\mathcal {G}}[\hat{{\varvec{\chi }}} \widehat{\varvec{E}}^{i}] + \varvec{\mathcal {G}}\left[ \hat{{\varvec{\chi }}}\varvec{\mathcal {G}}[\hat{{\varvec{\chi }}} \widehat{\varvec{E}}^{i}] \right] , \end{aligned}$$

or in coordinate writing

12 $$\begin{aligned} \begin{aligned} \widehat{\varvec{E}}^{2}(\omega ,\varvec{x})&= \widehat{\varvec{E}}^{i}(\omega ,\varvec{x})+ \frac{\omega ^2}{c^2}\int _{\mathbb {R}^3} \varvec{G}(\omega , \varvec{x}-\varvec{y})\hat{{\varvec{\chi }}}(\omega ,\varvec{y})\widehat{\varvec{E}}^{i}(\omega ,\varvec{y}){\,\mathrm d}\varvec{y} \\&\quad +\,\frac{\omega ^4}{c^4}\int _{\mathbb {R}^3} \int _{\mathbb {R}^3} \varvec{G}(\omega , \varvec{x}-\varvec{y})\hat{{\varvec{\chi }}}(\omega ,\varvec{y}) \varvec{G}(\omega , \varvec{y}-\varvec{z})\hat{{\varvec{\chi }}}(\omega ,\varvec{z}) \widehat{\varvec{E}}^{i}(\omega ,\varvec{z}){\,\mathrm d}\varvec{z}{\,\mathrm d}\varvec{y}, \end{aligned} \end{aligned}$$

where now $\varvec{G}$ is the Green tensor of Maxwell’s equations (Haddar 2004; Hazard and Lenoir 1996)

$$\begin{aligned} \varvec{G}(\omega , \varvec{x} - \varvec{y}) = G (\omega , \varvec{x} - \varvec{y}) \mathbb {1}+ \frac{c^2}{\omega ^2} \nabla \nabla \cdot (G (\omega , \varvec{x} - \varvec{y}) \mathbb {1}). \end{aligned}$$

The physical meaning of the second order Born-approximation is that at a point $\varvec{x}$ the total field $\widehat{\varvec{E}}^{2}$ contains all single and double scattering events.

In an OCT setup, the measurements are performed in a distance much bigger compared to the size of the sample. Thus, setting $\varvec{x} = \rho \varvec{\vartheta }, \rho >0$ and $\varvec{\vartheta }\in \mathbb {S}^2,$ we can replace the above expression by its asymptotic behaviour for $\rho \rightarrow \infty ,$ uniformly in $\varvec{\vartheta },$ see for instance Elbau et al. (2015, Equation (4.1)), resulting to

13 $$\begin{aligned} \widehat{\varvec{E}}^{2} (\omega ,\rho \varvec{\vartheta }) = \widehat{\varvec{E}}^{i}(\omega ,\rho \varvec{\vartheta })+ \varvec{\mathcal {G}}^\infty [\hat{{\varvec{\chi }}} \widehat{\varvec{E}}^{i}] (\omega ,\rho \varvec{\vartheta }) + \varvec{\mathcal {G}}^\infty \left[ \hat{{\varvec{\chi }}}\varvec{\mathcal {G}}[\hat{{\varvec{\chi }}} \widehat{\varvec{E}}^{i}] \right] (\omega , \rho \varvec{\vartheta }). \end{aligned}$$

Here we have introduced the operator

14 $$\begin{aligned} \varvec{\mathcal {G}}^\infty [\varvec{f} ] (\omega , \rho {\varvec{\vartheta }} ):= -\frac{\omega ^2 \mathrm{e}^{\mathrm{i}\tfrac{\omega }{c} \rho }}{4\pi \rho c^2} \int _{\mathbb {R}^3} \varvec{\vartheta }\times \left( \varvec{\vartheta }\times \varvec{f} (\omega ,\varvec{y}) \right) \mathrm{e}^{-\mathrm{i}\frac{\omega }{c}\left<\varvec{\vartheta },\varvec{y}\right>} {\,\mathrm d}\varvec{y}, \end{aligned}$$

defined for functions $\varvec{f}\,{:}\,\mathbb {R}\times \mathbb {R}^3 \rightarrow \mathbb {R}^3$.

## Polarized-sensitive OCT

We describe the standard PS-OCT system in the context of a Michelson interferometer first presented by Hee et al. (1992).

The detector array is given by $\mathcal D=\mathbb {R}^2\times \{d\}$ with $d>0$ sufficiently large. Moreover, we specify the CIF function to be ${\varvec{E}}^{0}$ as defined in Example 1 and we assume that ${\varvec{E}}^{0} (t,\varvec{x})=0$ for $t\ge 0$ and $\varvec{x}\in \mathcal D.$

We describe now the change in the polarization state of the light through the PS-OCT system. The effect of the polarization-insensitive beam splitter (BS) is not considered in this work since it only reduces the intensity of the beam by a constant factor. For simplicity, we place the sample and the mirror around the origin and the detector at the BS, for more details see Elbau et al. (2015, Section 3.3). The BS splits the light into two identical beams entering both arms of the interferometer.

**Fig. 2:**
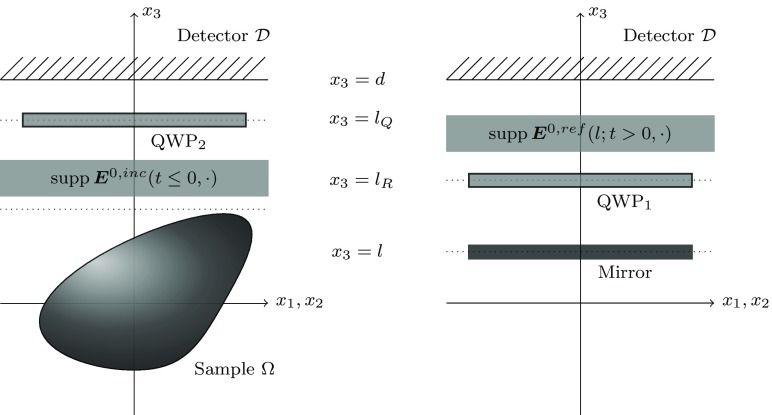
The two scattering problems in PS-OCT. On the left picture the incoming light in the sample arm passes through a QWP and is incident on the medium. On the right picture, in the reference arm, the light is back-reflected by a mirror (passing twice a QWP)

Reference arm: The light (at some negative time) passes through a zero-order quarter-wave plate (QWP) oriented at angle $\phi _1$ to the incident linear polarization. It is reflected by a perfect mirror placed in $x_3=l$ and then passes through the QWP again, at time $t = 0,$ see the right picture in Fig. 2. We formulate this process as a linear operator
  15 $$\begin{aligned} \varvec{\mathcal {J}}_l [{\varvec{E}}^{0} ] (t, \varvec{x}) = {\varvec{E}}^{0,{ ref}} (l;t,\varvec{x}), \end{aligned}$$
  to be specified later. Then, the reference field ${\varvec{E}}^l$ takes the form
  16 $$\begin{aligned} {\varvec{E}}^l(t,\varvec{x}) = {\left\{ \begin{array}{ll} {\varvec{E}}^{0} (t,\varvec{x}) + {\varvec{E}}^{0,{ ref}} (l;t,\varvec{x}), &{} \text {if }\,\, t> 0,\;\varvec{x}_3> l_R,\\ {\varvec{E}}^{0}(t,\varvec{x}), &{} \text {if }\,\, t\le 0,\;\varvec{x}_3 > l_R. \end{array}\right. } \end{aligned}$$
Sample arm: The incoming light passes through a QWP (oriented at a different angle $\phi _2 )$ at some time $t<0,$ placed in the plane given by the equation $x_3 = l_Q.$ This process results to a field
  17 $$\begin{aligned} \varvec{\mathcal {J}} [{\varvec{E}}^{0} ] (t, \varvec{x}) = {\varvec{E}}^{0,{ inc}} (t,\varvec{x}), \end{aligned}$$
  that until $t = 0$ does not interact with the medium, see the left picture in Fig. 2.
Detector: The electric field ${\varvec{E}}$ which is obtained by illuminating the sample with the incident field ${\varvec{E}}^{0,{ inc}}$ is combined with the reference field ${\varvec{E}}^l.$ We assume here that the backscattered light does not pass through the QWP again. At every point on the detector surface $\mathcal D$ we measure the two intensities (Elbau et al. 2015)
  $$\begin{aligned}&I_j(l,\varvec{\xi })=\int _{0}^\infty E_j (t,\varvec{\xi }) E^l_j (t,\varvec{\xi }){\,\mathrm d}t,\quad \varvec{\xi }\in \mathcal D,\;j\in \{1,2\}. \end{aligned}$$

We assume that we do not measure the incident fields at the detector, meaning ${\varvec{E}}^{0}(t,\varvec{\xi })={\varvec{E}}^{0,{ inc}}(t,\varvec{\xi })=0$ for $t> 0$ and $\varvec{\xi }\in \mathcal D$ and recalling (16) we obtain $\varvec{E}^l-\varvec{E}^0 = 0$ for $t\le 0,$ resulting to

18 $$\begin{aligned} I_j(l,\varvec{\xi })&= \int _{0}^\infty (E_j- E^{0,{ inc}}_j)(t,\varvec{\xi })(E^l_j -E^{0}_j)(t,\varvec{\xi }){\,\mathrm d}t\nonumber \\&= \int _{\mathbb {R}}(E_j- E^{0,{ inc}}_j)(t,\varvec{\xi })(E^l_j -E_j^{0})(t,\varvec{\xi }){\,\mathrm d}t. \end{aligned}$$

We use Plancherel’s theorem, and since ${\varvec{E}}\in \mathbb {R}^3$ it follows that $\widehat{\varvec{E}}(-\omega ,\cdot ) = \overline{\widehat{\varvec{E}}}(\omega ,\cdot ).$ Thus, the above formula can be rewritten as

19 $$\begin{aligned} I_j(l,\varvec{\xi })&= \frac{1}{2\pi }\int _{\mathbb {R}}(\hat{E}_j-\hat{E}_j^{0,{ inc}})(\omega ,\varvec{\xi })(\overline{\hat{E}^l_j -\hat{E}_j^{0}})(\omega ,\varvec{\xi }){\,\mathrm d}\omega \nonumber \\&= \frac{1}{2\pi }\int _{-\infty }^0 (\overline{\hat{E}_j-\hat{E}_j^{0,{ inc}}})(-\omega ,\varvec{\xi })(\hat{E}^l_j -\hat{E}_j^{0})(-\omega ,\varvec{\xi }){\,\mathrm d}\omega \nonumber \\&\quad +\,\frac{1}{2\pi }\int _0^\infty (\hat{E}_j-\hat{E}_j^{0,{ inc}})(\omega ,\varvec{\xi })(\overline{\hat{E}^l_j -\hat{E}_j^{0}})(\omega ,\varvec{\xi }){\,\mathrm d}\omega \nonumber \\&= \frac{1}{\pi } \mathfrak {R}\int _0^\infty (\hat{E}_j-\hat{E}_j^{0,{ inc}})(\omega ,\varvec{\xi })(\overline{\hat{E}^l_j -\hat{E}_j^{0}})(\omega ,\varvec{\xi }){\,\mathrm d}\omega . \end{aligned}$$

### Jones calculus

Here we describe the operators $\varvec{\mathcal {J}}_l$ and $\varvec{\mathcal {J}}$, introduced in (15) and (17), respectively. We consider the fields in the frequency domain. Then, for positive frequencies we can apply the Jones matrix method (keeping also the zero third component of the fields) in order to model the effect of the QWP’s on the polarization state of light. We assume that the properties of the QWP’s are frequency independent and that the light is totally transmitted through the plate surface.

#### Definition 4

We define

20 $$\begin{aligned} \begin{aligned} \varvec{\mathcal {J}}_l [\varvec{v} ] (\omega , \varvec{x})&= \varvec{J}^2 (\phi _1 ) \varvec{v} (\omega ,\varvec{x}) \, \mathrm{e}^{\mathrm{i}\tfrac{\omega }{c} 2(x_3 - l)},&\quad \text{ for } \,\, \omega> 0,\\ \varvec{\mathcal {J}} [\varvec{v} ] (\omega , \varvec{x})&= \varvec{J} (\phi _2) \varvec{v} (\omega ,\varvec{x}),&\quad \text{ for } \,\, \omega > 0, \end{aligned} \end{aligned}$$

where

$$\begin{aligned} \varvec{J} (\phi )= \begin{pmatrix} \cos \phi &{}\quad -\sin \phi &{}\quad 0\\ \sin \phi &{}\quad \phantom {-}\cos \phi &{}\quad 0 \\ 0 &{}\quad 0 &{}\quad 1 \end{pmatrix} \begin{pmatrix} 1 &{}\quad \phantom {-}0 &{}\quad 0 \\ 0 &{}\quad -\mathrm{i}&{}\quad 0 \\ 0 &{}\quad \phantom {-}0 &{}\quad 1 \end{pmatrix} \begin{pmatrix} \phantom {-}\cos \phi &{}\quad \sin \phi &{}\quad 0\\ -\sin \phi &{}\quad \cos \phi &{}\quad 0 \\ 0 &{}\quad 0 &{}\quad 1 \end{pmatrix}, \end{aligned}$$

is the rotated Jones matrix for a QWP with the fast axis oriented at angle $\phi$ (Gerrard and Burch 1975).

The above definition summarizes what we described before: In the reference arm, the incoming field passes through the QWP (at angle $\phi _1)$ is reflected by the mirror and then passes through the QWP again. The field travels additionally the distance $2(x_3 - l).$ In the sample arm, the field passes only through the QWP at angle $\phi _2.$

We consider the PS-OCT system, presented first by Hee et al. (1992) and then considered by Hitzenberger et al. (2001) and Schoenenberger et al. (1998), where $\phi _1 = \pi /8$ and $\phi _2 = \pi /4.$ Then, we obtain

21 $$\begin{aligned} \begin{aligned} \widehat{\varvec{E}}^{0,{ ref}} (l;\omega ,\varvec{x})&= \varvec{\mathcal {J}}_l [\widehat{\varvec{E}}^{0} ] (\omega , \varvec{x}) = \varvec{\eta }\hat{f}(\omega ) \,\mathrm{e}^{\mathrm{i}\tfrac{\omega }{c} (x_3 - 2l)},&\text{ for } \omega> 0,\\ \widehat{\varvec{E}}^{0,{ inc}} (\omega ,\varvec{x})&= \varvec{\mathcal {J}} [\widehat{\varvec{E}}^{0} ] (\omega , \varvec{x}) = \varvec{p} \hat{f}(\omega ) \, \mathrm{e}^{-\mathrm{i}\tfrac{\omega }{c} x_3 },&\text{ for } \omega > 0,\\ \end{aligned} \end{aligned}$$

where $\varvec{\eta }= \varvec{J}^2 (\pi /8) \, \varvec{q}$ and $\varvec{p} = \varvec{J} (\pi /4) \, \varvec{q}.$ We observe that $\widehat{\varvec{E}}^{0,{ ref}}$ is still linearly polarized at angle $\pi /4$ with the linear (horizontal or vertical) initial polarization state and $\widehat{\varvec{E}}^{0,{ inc}}$ describes a circularly polarized light.

Now we can define our approximated data. We approximate in (19) the term $\hat{E}_j-\hat{E}_j^{0,{ inc}}$ by $\hat{E}^{2}_j-\hat{E}_j^{0,{ inc}}$ and for the term $\hat{E}^l_j -\hat{E}_j^{0}$ we consider (16) and (21).

#### Definition 5

We call

22 $$\begin{aligned} I_j^{2}(l,\varvec{\xi }) = \frac{\eta _j}{\pi } \mathfrak {R}\int _0^\infty (\hat{E}^{2}_j-\hat{E}_j^{0,{ inc}})(\omega ,\varvec{\xi }) \hat{f}(-\omega ) \mathrm{e}^{\mathrm{i}\tfrac{\omega }{c} (2l-\xi _3)}{\,\mathrm d}\omega . \end{aligned}$$

the second order approximated measurement data of OCT.

## The inverse problem of recovering the susceptibility

The problem we address here, is to recover $\hat{{\varvec{\chi }}}$ from the knowledge of $\varvec{I}^2 (l,\varvec{\xi })$ for $l\in \mathbb {R}, \varvec{\xi }\in \mathcal D.$ First, we show that the measurements provide us with expressions which depend on $\hat{{\varvec{\chi }}}$ non-linearly.

### Proposition 1

Let ${\varvec{E}}^{0}(t,\varvec{x})$ be given by the form (6) with $q_3=0$ and let the measurement data $I^2_j$ be given by (22). Then, for every $\omega \in \mathbb {R}_+{\setminus }\{0\}$ with $\hat{f}(\omega )\ne 0$, the expression

23 $$\begin{aligned}&\eta _j \left[ \varvec{\mathcal {G}}^\infty \left[ \hat{{\varvec{\chi }}}\left( \varvec{p} \, \mathrm{e}^{-\mathrm{i}\frac{\omega }{c} y_3} + \varvec{\mathcal {G}}[\hat{{\varvec{\chi }}} \varvec{p} \, \mathrm{e}^{-\mathrm{i}\frac{\omega }{c} z_3} ] \right) \right] \right] _j (\omega , \rho {\varvec{\vartheta }} ) \nonumber \\&\quad = \frac{1}{ c | \hat{f}(\omega )|^2 }\int _{\mathbb {R}} I^2_j( l,\rho {\varvec{\vartheta }})\mathrm{e}^{-\mathrm{i}\frac{\omega }{c}(2l-\rho \vartheta _3)}{\,\mathrm d}l \end{aligned}$$

holds for all $j\in \{1,2\}$, and ${\varvec{\vartheta }}\in \mathbb {S}^2_+ :=\{{\varvec{\mu }}\in \mathbb {S}^2\mid \mu _3>0\}.$

### Proof

We consider Eq. (13) where now $\widehat{\varvec{E}}^{i}$ is replaced by $\widehat{\varvec{E}}^{0,{ inc}}$ for $\omega >0$. Then, we get

24 $$\begin{aligned} (\widehat{\varvec{E}}^{2}-\widehat{\varvec{E}}^{0,{ inc}})(\omega ,\rho {\varvec{\vartheta }}) = \hat{f}(\omega )\, \varvec{\mathcal {G}}^\infty \left[ \hat{{\varvec{\chi }}}\left( \varvec{p} \, \mathrm{e}^{-\mathrm{i}\frac{\omega }{c} y_3} + \varvec{\mathcal {G}}[\hat{{\varvec{\chi }}} \varvec{p} \, \mathrm{e}^{-\mathrm{i}\frac{\omega }{c} z_3} ] \right) \right] (\omega , \rho \varvec{\vartheta }). \end{aligned}$$

We apply the inverse Fourier transform with respect to *l* in (22), to obtain

25 $$\begin{aligned} \begin{aligned} \int _\mathbb {R}I_j^{2}(l,\varvec{\xi }) \mathrm{e}^{-\mathrm{i}\tfrac{\tilde{\omega }}{c}2l} {\,\mathrm d}l&= \frac{c\eta _j}{2} \int _0^\infty (\hat{E}^{2}_j-\hat{E}_j^{0,{ inc}})(\omega ,\varvec{\xi }) \hat{f}(-\omega ) \mathrm{e}^{-\mathrm{i}\tfrac{\omega }{c} \xi _3 } \delta (\omega - \tilde{\omega }){\,\mathrm d}\omega \\&\phantom {=}\quad +\,\frac{c\eta _j}{2} \int _0^\infty \overline{(\hat{E}^{2}_j-\hat{E}_j^{0,{ inc}})(\omega ,\varvec{\xi }) \hat{f}(-\omega ) \mathrm{e}^{-\mathrm{i}\tfrac{\omega }{c} \xi _3 }} \delta (\omega + \tilde{\omega }){\,\mathrm d}\omega \end{aligned} \end{aligned}$$

which for $\tilde{\omega }>0, \,\hat{f} \ne 0$ and $\eta _j \ne 0,$ using that $\varvec{E}$ and *f* are real valued, results to

$$\begin{aligned} (\hat{E}^{2}_j-\hat{E}_j^{0,{ inc}})(\omega ,\varvec{\xi }) = \frac{1}{ \eta _j c \hat{f}(-\omega ) }\int _{\mathbb {R}} I^2_j( l,\varvec{\xi })\mathrm{e}^{-\mathrm{i}\frac{\omega }{c}(2l-\xi _3)}{\,\mathrm d}l. \end{aligned}$$

This identity together with (24), results asymptotically to (23). $\square$

We observe here that we want to reconstruct four four-dimensional functions from two three-dimensional measurement data. Thus, we have to consider some additional assumptions on the medium in order to cancel out the lack of dimensions and handle the non-linearity of (23) with respect to $\hat{{\varvec{\chi }}}.$

### Assumption 1

Specific illumination: The support of the initial pulse is small enough such that the optical parameters in this spectrum can be assumed constant with respect to frequency.

Medium: The susceptibility can be decomposed into two parts, a background susceptibility which is constant and assumed to be known and a part that counts for the local variations of the susceptibility and can be seen as deviation from the constant value.

Then, the expression (3) admits the special form

$$\begin{aligned} \hat{{\varvec{\chi }}} (\omega , \varvec{x})= {\varvec{\chi }}_0 + \epsilon \,{\varvec{\psi }} (\varvec{x}), \end{aligned}$$

where

$$\begin{aligned} {\varvec{\chi }}_0 = \chi _0 \begin{pmatrix} 1 &{}\quad 1 &{}\quad 0 \\ 1 &{}\quad 1 &{}\quad 0 \\ 0 &{}\quad 0 &{}\quad 1 \end{pmatrix}, \quad \text{ and } \quad {\varvec{\psi }} = \begin{pmatrix} \psi _{11} &{}\quad \psi _{12} &{}\quad 0 \\ \psi _{12} &{}\quad \psi _{22} &{}\quad 0 \\ 0 &{}\quad 0 &{}\quad \psi _{33} \end{pmatrix}, \end{aligned}$$

for some known $\chi _0 \in \mathbb {R}$, a small parameter $\epsilon >0$ and $\psi _{ij} \in C^\infty _{\mathrm c}(\mathbb {R}^3; \mathbb {C}).$

In the following, we consider this type of media, which is typical for biological tissues, and we assume in addition that the behavior of the homogeneous medium ($\epsilon = 0$) is known. Then, as a consequence, also the measured data from PS-OCT are known, let us call them $I_0$, and we can assume the following form for the measurements

26 $$\begin{aligned} I^2_j(l,\varvec{\xi }) = I_0 + \epsilon M_j(l,\varvec{\xi }), \quad l \in \mathbb {R}, \, \varvec{\xi }\in \mathcal {D}, \, j\in \{1,2\}, \end{aligned}$$

for some known functions $M_j.$

### Proposition 2

Let the assumptions of Proposition 1 and the additional Assumption 1 hold. We define $\varvec{v}= \tfrac{\omega }{c} (\varvec{\vartheta }+\varvec{e}_3),\, {\varvec{\vartheta }}\in \mathbb {S}^2_+.$ Then, the spatial Fourier transform of the matrix-valued function $\varvec{\psi }: \mathbb {R}^3 \rightarrow \mathbb {C}^{3\times 3},$ satisfies the equations

27 $$\begin{aligned} \eta _j \left[ \varvec{\vartheta }\times \left( \varvec{\vartheta }\times \left( \left( \varvec{\tilde{\psi }}(\varvec{v}) + {\varvec{\chi }}_0 \varvec{\mathcal {K}} [\varvec{\tilde{\psi }}] (\varvec{v}) + \varvec{\mathcal {K}}^\dagger [\varvec{\tilde{\psi }}] (\varvec{v}) {\varvec{\chi }}_0 \right) \varvec{p} \right) \right) \right] _j = \tilde{m}_j (\varvec{v} ), \quad j\in \{1,2\}, \end{aligned}$$

where

28 $$\begin{aligned} \tilde{m}_j (\varvec{v} ):= m_j (\omega , \varvec{\vartheta }) = -\frac{4\pi \rho c}{ \omega ^2| \hat{f}(\omega )|^2 }\int _{\mathbb {R}} M_j( l,\rho {\varvec{\vartheta }})\mathrm{e}^{-\mathrm{i}\frac{\omega }{c}(2l-\rho (\vartheta _3-1))}{\,\mathrm d}l. \end{aligned}$$

The operators $\varvec{\mathcal {K}}$ and $\varvec{\mathcal {K}}^\dagger$ are defined by

29 $$\begin{aligned} \varvec{\mathcal {K}} [\varvec{f} ] (\varvec{v}): = \int _{\mathbb {R}^3} \varvec{K}^{\varvec{z}} (\varvec{v}; \varvec{k}) \varvec{f} (\varvec{k}) {\,\mathrm d}\varvec{k}, \quad \varvec{\mathcal {K}}^\dagger [\varvec{f} ] (\varvec{v}): = \int _{\mathbb {R}^3} \varvec{f} (\varvec{k}) \varvec{K}^{\varvec{y}} (\varvec{v}; \varvec{k}) {\,\mathrm d}\varvec{k}, \end{aligned}$$

for functions $\varvec{f}\,{:}\,\mathbb {R}^3 \rightarrow \mathbb {C}^{3\times 3},$ with kernels

$$\begin{aligned} \varvec{K}^{\varvec{\alpha }} (\tfrac{\omega }{c} (\varvec{\vartheta }+\varvec{e}_3); \varvec{k}) = \frac{\omega ^2 }{c^2(2\pi )^3} \int _{\varOmega }\int _{\varOmega } \varvec{G}(\omega , \varvec{y}-\varvec{z})\mathrm{e}^{-\mathrm{i}\frac{\omega }{c} (z_3 + \left<\varvec{\vartheta },\varvec{y}\right>)} \mathrm{e}^{\mathrm{i}\left<\varvec{k},\varvec{\alpha }\right>} {\,\mathrm d}\varvec{z}{\,\mathrm d}\varvec{y}, \end{aligned}$$

for $$\varvec{\alpha }= \varvec{z}, \varvec{y}.$$

### Proof

We substitute $\hat{{\varvec{\chi }}},$ considering Assumption 1, and (26) in (23) and we equate the first order terms $\varvec{\psi }$ and $\varvec{M}$ to obtain

30 $$\begin{aligned}&\eta _j \left[ \varvec{\mathcal {G}}^\infty \left[ \varvec{\psi }\left( \varvec{p} \, \mathrm{e}^{-\mathrm{i}\frac{\omega }{c} y_3} + \varvec{\mathcal {G}}\left[ {\varvec{\chi }}_0 \varvec{p} \mathrm{e}^{-\mathrm{i}\frac{\omega }{c} z_3}\right] \right) \right] \right] _j (\omega , \rho {\varvec{\vartheta }} ) \nonumber \\&\qquad +\,\eta _j \left[ \varvec{\mathcal {G}}^\infty \left[ {\varvec{\chi }}_0 \varvec{\mathcal {G}}\left[ \varvec{\psi }\varvec{p} \, \mathrm{e}^{-\mathrm{i}\frac{\omega }{c} z_3 }\right] \right] \right] _j (\omega , \rho {\varvec{\vartheta }} ) \nonumber \\&\quad = \frac{1}{ c | \hat{f}(\omega )|^2 }\int _{\mathbb {R}} M_j( l,\rho {\varvec{\vartheta }})\mathrm{e}^{-\mathrm{i}\frac{\omega }{c}(2l-\rho \vartheta _3)}{\,\mathrm d}l. \end{aligned}$$

In order to analyse the left hand side of the above equation we consider the definition (14) and the analytic form (12). Then, we rewrite (30) as

$$\begin{aligned}&\eta _j \left[ \int _{\varOmega } \varvec{\vartheta }\times \left( \varvec{\vartheta }\times \left( {\varvec{\psi }} (\varvec{y})\varvec{p}\right) \right) \mathrm{e}^{-\mathrm{i}\frac{\omega }{c}\left<\varvec{\vartheta }+\varvec{e}_3,\varvec{y}\right>} {\,\mathrm d}\varvec{y} \right. \\&\qquad +\, \frac{\omega ^2 }{c^2} \int _{\varOmega } \int _{\varOmega } \varvec{\vartheta }\times \left( \varvec{\vartheta }\times \left( {\varvec{\chi }}_0 \varvec{G}(\omega , \varvec{y}-\varvec{z}){\varvec{\psi }} (\varvec{z}) \varvec{p} \right) \right) \mathrm{e}^{-\mathrm{i}\frac{\omega }{c} (z_3 + \left<\varvec{\vartheta },\varvec{y}\right>)} {\,\mathrm d}\varvec{z}{\,\mathrm d}\varvec{y} \\&\qquad +\, \left. \frac{\omega ^2 }{c^2} \int _{\varOmega } \int _{\varOmega } \varvec{\vartheta }\times \left( \varvec{\vartheta }\times \left( {\varvec{\psi }} (\varvec{y}) \varvec{G}(\omega , \varvec{y}-\varvec{z}){\varvec{\chi }}_0 \varvec{p}\right) \right) \mathrm{e}^{-\mathrm{i}\frac{\omega }{c} (z_3 + \left<\varvec{\vartheta },\varvec{y}\right>)} {\,\mathrm d}\varvec{z}{\,\mathrm d}\varvec{y} \right] _j \\&\quad = m_j (\omega ,\varvec{\vartheta }), \end{aligned}$$

where $m_j$ is given by (28). Taking the Fourier transform of ${\varvec{\psi }}$ with respect to space, we get

31 $$\begin{aligned}&\eta _j \left[ \varvec{\vartheta }\times \left( \varvec{\vartheta }\times \left( \varvec{\tilde{\psi }}(\tfrac{\omega }{c} (\varvec{\vartheta }+\varvec{e}_3)) \varvec{p} \right) \right) \right. \nonumber \\&\qquad +\, \frac{\omega ^2 }{c^2(2\pi )^3} \int _{\mathbb {R}^3}\int _{\varOmega } \int _{\varOmega } \varvec{\vartheta }\times \left( \varvec{\vartheta }\times \left( {\varvec{\chi }}_0 \varvec{G}(\omega , \varvec{y}-\varvec{z}) \varvec{\tilde{\psi }}(\varvec{k}) \varvec{p}\right) \right) \nonumber \\&\qquad \times \mathrm{e}^{-\mathrm{i}\frac{\omega }{c} (z_3 + \left<\varvec{\vartheta },\varvec{y}\right>)}\mathrm{e}^{\mathrm{i}\left<\varvec{k},\varvec{z}\right>} {\,\mathrm d}\varvec{z}{\,\mathrm d}\varvec{y} {\,\mathrm d}\varvec{k} \nonumber \\&\qquad + \frac{\omega ^2 }{c^2(2\pi )^3} \int _{\mathbb {R}^3}\int _{\varOmega } \int _{\varOmega } \varvec{\vartheta }\times \left( \varvec{\vartheta }\times \left( \varvec{\tilde{\psi }}(\varvec{k}) \varvec{G}(\omega , \varvec{y}-\varvec{z}) {\varvec{\chi }}_0 \varvec{p}\right) \right) \mathrm{e}^{-\mathrm{i}\frac{\omega }{c} (z_3 + \left<\varvec{\vartheta },\varvec{y}\right>)} \nonumber \\&\qquad \left. \times \,\mathrm{e}^{\mathrm{i}\left<\varvec{k},\varvec{y}\right>} {\,\mathrm d}\varvec{z}{\,\mathrm d}\varvec{y} {\,\mathrm d}\varvec{k} \right] _j = m_j (\omega ,\varvec{\vartheta }). \end{aligned}$$

This equation for $\varvec{\tilde{m}} (\varvec{v} ):= \varvec{m} (\omega ,\varvec{\vartheta }),$ using the definitions of the integral operators (29) admits the compact form (27). $\square$

Regarding the integral operators appearing in (29), we prove the following property.

### Lemma 2

The integral operators $\varvec{\mathcal {K}}, \, \varvec{\mathcal {K}}^\dagger : (L^2 (\varOmega ))^{3\times 3} \rightarrow (L^2 (\mathbb {S}^2))^{3\times 3},$ defined by (29), are compact.

### Proof

We consider the following decomposition

$$\begin{aligned} \varvec{\mathcal {K}} [\varvec{f} ] (\tfrac{\omega }{c} (\varvec{\vartheta }+\varvec{e}_3))= & {} \frac{\omega ^2 }{c^2(2\pi )^3} \int _{\mathbb {R}^3}\int _{\varOmega } \int _{\varOmega } \varvec{G}(\omega , \varvec{y}-\varvec{z})\\&\times \mathrm{e}^{-\mathrm{i}\frac{\omega }{c} (z_3 + \left<\varvec{\vartheta },\varvec{y}\right>)} \mathrm{e}^{\mathrm{i}\left<\varvec{k},\varvec{z}\right>} \varvec{\tilde{f}} (\varvec{k}) {\,\mathrm d}\varvec{z}{\,\mathrm d}\varvec{y} {\,\mathrm d}\varvec{k}\\= & {} \frac{\omega ^2 }{c^2} \int _{\varOmega } \int _{\varOmega } \varvec{G}(\omega , \varvec{y}-\varvec{z})\mathrm{e}^{-\mathrm{i}\frac{\omega }{c} (z_3 + \left<\varvec{\vartheta },\varvec{y}\right>)} \varvec{f} (\varvec{z}) {\,\mathrm d}\varvec{z}{\,\mathrm d}\varvec{y} \\= & {} \int _{\varOmega } \mathrm{e}^{-\mathrm{i}\frac{\omega }{c} \left<\varvec{\vartheta },\varvec{y}\right>} \left( \frac{\omega ^2}{c^2} \mathbb {1}\int _{\varOmega }G(\omega , \varvec{y}-\varvec{z}) \mathrm{e}^{-\mathrm{i}\frac{\omega }{c} z_3} \varvec{f} (\varvec{z}) {\,\mathrm d}\varvec{z} \right. \\&\left. +\,\nabla \nabla \cdot \int _{\varOmega }G(\omega , \varvec{y}-\varvec{z}) \mathrm{e}^{-\mathrm{i}\frac{\omega }{c} z_3} \varvec{f} (\varvec{z}) {\,\mathrm d}\varvec{z} \right) {\,\mathrm d}\varvec{y} \\= & {} \int _{\varOmega } \mathrm{e}^{-\mathrm{i}\frac{\omega }{c} \left<\varvec{\vartheta },\varvec{y}\right>} \left( \frac{\omega ^2}{c^2} \mathbb {1}\int _{\varOmega }G(\omega , \varvec{y}-\varvec{z}) \mathrm{e}^{-\mathrm{i}\frac{\omega }{c} z_3} \varvec{f} (\varvec{z}) {\,\mathrm d}\varvec{z} \right. \\&\left. +\,\nabla \nabla \cdot \int _{\varOmega }G(0, \varvec{y}-\varvec{z}) \mathrm{e}^{-\mathrm{i}\frac{\omega }{c} z_3} \varvec{f} (\varvec{z}) {\,\mathrm d}\varvec{z} \right. \\&\left. +\,\nabla \nabla \cdot \int _{\varOmega }\left( G(\omega , \varvec{y}-\varvec{z}) - G(0, \varvec{y}-\varvec{z})\right) \mathrm{e}^{-\mathrm{i}\frac{\omega }{c} z_3} \varvec{f} (\varvec{z}) {\,\mathrm d}\varvec{z} \right) {\,\mathrm d}\varvec{y}. \end{aligned}$$

The above expression in compact form reads

$$\begin{aligned} \varvec{\mathcal {K}} [\varvec{f} ] (\varvec{v}) = \varvec{\mathcal {F}} \left[ (\mathcal {G} +\varvec{\mathcal {G}}_0+\varvec{\mathcal {G}}_1) [\mathrm{e}^{-\mathrm{i}\frac{\omega }{c} z_3}\varvec{f}] \right] (\varvec{v}), \end{aligned}$$

for the operators

$$\begin{aligned} \begin{aligned} \varvec{\mathcal {F}} [ f ] (\varvec{\theta })&:= \int _{\varOmega } \mathrm{e}^{-\mathrm{i}\frac{\omega }{c} \left<\varvec{\vartheta },\varvec{y}\right>} f (\varvec{y}) {\,\mathrm d}\varvec{y},\\ \mathcal {G} [ f ] (\varvec{x})&:= \frac{\omega ^2}{c^2} \int _{\varOmega }G(\omega , \varvec{x}-\varvec{y}) f (\varvec{y}) {\,\mathrm d}\varvec{y},\\ \varvec{\mathcal {G}}_0 [\varvec{f} ] (\varvec{x})&:= \nabla \nabla \cdot \int _{\varOmega }G(0, \varvec{x}-\varvec{y}) \varvec{f} (\varvec{y}) {\,\mathrm d}\varvec{y},\\ \varvec{\mathcal {G}}_1 [\varvec{f} ] (\varvec{x})&:= \nabla \nabla \cdot \int _{\varOmega }\left( G(\omega , \varvec{x}-\varvec{y}) - G(0, \varvec{x}-\varvec{y})\right) \varvec{f} (\varvec{y}) {\,\mathrm d}\varvec{y}. \end{aligned} \end{aligned}$$

The operator $\varvec{\mathcal {F}}: L^2 (\varOmega ) \rightarrow L^2 (\mathbb {S}^2)$ is a modification of the usual far-field operator with smooth kernel, thus compact. The operators $\mathcal {G}: L^2 (\varOmega ) \rightarrow L^2 (\varOmega )$ and $\varvec{\mathcal {G}}_1\,{:}\,(L^2 (\varOmega ))^{3\times 3} \rightarrow (L^2 (\varOmega ))^{3\times 3}$ are also compact due to their weakly singular kernels, see for instance (Colton and Kress 2013; Potthast 2000), and the operator $\varvec{\mathcal {G}}_0\,{:}\,(L^2 (\varOmega ))^{3\times 3} \rightarrow (L^2 (\varOmega ))^{3\times 3}$ is bounded (Colton et al. 2007). Thus $\varvec{\mathcal {K}}\,{:}\,(L^2 (\varOmega ))^{3\times 3} \rightarrow (L^2 (\mathbb {S}^2))^{3\times 3}$ is also compact. The same arguments hold also for the operator $\varvec{\mathcal {K}}^\dagger . \square$

Now, we are in position to formulate the inverse problem: Recover from the expressions

$$\begin{aligned} \eta _j \left[ \varvec{\vartheta }\times \left( \varvec{\vartheta }\times \left( \left( \varvec{\tilde{\psi }}(\varvec{v}) + {\varvec{\chi }}_0 \varvec{\mathcal {K}} [\varvec{\tilde{\psi }}] (\varvec{v}) + \varvec{\mathcal {K}}^\dagger [\varvec{\tilde{\psi }}] (\varvec{v}) {\varvec{\chi }}_0 \right) \varvec{p} \right) \right) \right] _j, \quad j\in \{1,2\}, \end{aligned}$$

the matrix-valued function $\varvec{\psi }\,{:}\,\varOmega \rightarrow \mathbb {C}^{3\times 3},$ assuming that we have measurements for every incident polarization.

Let us now specify the polarization vectors $\varvec{\eta }$ and $\varvec{p}.$ We choose two different incident polarization vectors $\varvec{q}^{(1)} = \varvec{e}_1$ and $\varvec{q}^{(2)} = \varvec{e}_2,$ and using the formulas (21) we obtain the vectors

32 $$\begin{aligned} \begin{aligned} \varvec{\eta }^{(1)}&= \frac{\sqrt{2}}{2} \begin{pmatrix} 1 \\ 1 \\ 0 \end{pmatrix},&\varvec{p}^{(1)}&= \frac{1}{2} \begin{pmatrix} 1 -\mathrm{i}\\ 1 +\mathrm{i}\\ 0 \end{pmatrix}, \\ \varvec{\eta }^{(2)}&= \frac{\sqrt{2}}{2} \begin{pmatrix} \phantom {-}1 \\ -1 \\ \phantom {-}0 \end{pmatrix},&\varvec{p}^{(2)}&= \frac{1}{2} \begin{pmatrix} 1 +\mathrm{i}\\ 1 -\mathrm{i}\\ 0 \end{pmatrix}. \end{aligned} \end{aligned}$$

### Remark 1

To find, for instance, the form of the incident wave $\varvec{p}^{(1)} \hat{f}(\omega ) \, \mathrm{e}^{-\mathrm{i}\tfrac{\omega }{c} x_3 },$ for $\omega >0,$ in the time domain we have to extend it for negative frequencies and consider its inverse Fourier transform. Then, we have

$$\begin{aligned} \begin{aligned} \varvec{E}^{(1)} (t,\varvec{x})&=: \frac{1}{2\pi } \int _0^\infty \varvec{p}^{(1)} \hat{f}(\omega ) \, \mathrm{e}^{-\mathrm{i}\tfrac{\omega }{c} x_3 } \mathrm{e}^{-\mathrm{i}\omega t} {\,\mathrm d}\omega \\&\quad +\,\frac{1}{2\pi } \int _{-\infty }^0 \overline{\varvec{p}^{(1)}} \hat{f}(\omega ) \, \mathrm{e}^{-\mathrm{i}\tfrac{\omega }{c} x_3 } \mathrm{e}^{-\mathrm{i}\omega t} {\,\mathrm d}\omega \\&= \frac{1}{2\pi } \int _0^\infty \varvec{p}^{(1)} \hat{f}(\omega ) \, \mathrm{e}^{-\mathrm{i}\tfrac{\omega }{c} x_3 } \mathrm{e}^{-\mathrm{i}\omega t} {\,\mathrm d}\omega \\&\quad +\,\frac{1}{2\pi } \int _0^{\infty } \overline{\varvec{p}^{(1)} \hat{f}(\omega ) \, \mathrm{e}^{-\mathrm{i}\tfrac{\omega }{c} x_3 } \mathrm{e}^{-\mathrm{i}\omega t}} {\,\mathrm d}\omega \\&=\frac{1}{\pi } \mathfrak {R}\int _0^\infty \varvec{p}^{(1)} \hat{f}(\omega ) \, \mathrm{e}^{-\mathrm{i}\tfrac{\omega }{c} x_3 } \mathrm{e}^{-\mathrm{i}\omega t} {\,\mathrm d}\omega \end{aligned} \end{aligned}$$

If the small spectrum is centered around a frequency $\nu ,$ we approximate $\hat{f}(\omega ) \simeq \delta (\omega - \nu ),$ for $\omega >0,$ to obtain

$$\begin{aligned} \begin{aligned} \varvec{E}^{(1)} (t,\varvec{x})&= \frac{1}{\pi } \mathfrak {R}\left\{ \varvec{p}^{(1)} \mathrm{e}^{-\mathrm{i}\nu (\tfrac{x_3}{c}+t) } \right\} \\&= \frac{1}{2\pi } \begin{pmatrix} \cos (\nu (\tfrac{x_3}{c}+t)) - \sin (\nu (\tfrac{x_3}{c}+t)) \\ \cos (\nu (\tfrac{x_3}{c}+t)) + \sin (\nu (\tfrac{x_3}{c}+t)) \\ 0 \end{pmatrix} \\&= \frac{1}{\sqrt{2}\pi } \begin{pmatrix} \cos (\tfrac{\pi }{4} +\nu (\tfrac{x_3}{c}+t)) \\ \sin (\tfrac{\pi }{4} +\nu (\tfrac{x_3}{c}+t)) \\ 0 \end{pmatrix}. \end{aligned} \end{aligned}$$

We see that $\varvec{E}^{(1)}$ describes also a circularly polarized wave with a phase shift.

If we neglect the zeroth third components, we observe that $\varvec{\eta }^{(1)},\varvec{\eta }^{(2)} \in \mathbb {R}^2$ and $\varvec{p}^{(1)}, \varvec{p}^{(2)} \in \mathbb {C}^2$ form a basis in $\mathbb {R}^2$ and $\mathbb {C}^2,$ respectively. The following result shows that measurements for additional polarization vectors $\varvec{q}$ do not provide any further information.

### Proposition 3

Let $\varvec{\vartheta }\in \mathbb {S}^2_+$ be fixed and $\varvec{q} = \varvec{q}^{(1)}, \, \varvec{q}^{(2)}.$ Then, the Eq. (27) is equivalent to the system of equations

33 $$\begin{aligned} \begin{aligned} \left[ \varvec{P}_{\varvec{\vartheta }} \varvec{Y} \varvec{p}^{(1)}\right] _1&= b^{(1)}_1,\quad&\left[ \varvec{P}_{\varvec{\vartheta }} \varvec{Y} \varvec{p}^{(1)}\right] _2&= b^{(1)}_2,\\ \left[ \varvec{P}_{\varvec{\vartheta }} \varvec{Y} \varvec{p}^{(2)}\right] _1&= b^{(2)}_1,\quad&\left[ \varvec{P}_{\varvec{\vartheta }} \varvec{Y} \varvec{p}^{(2)}\right] _2&= -b^{(2)}_2, \end{aligned} \end{aligned}$$

where $\varvec{Y}:= \varvec{\tilde{\psi }}(\varvec{v}) + {\varvec{\chi }}_0 \varvec{\mathcal {K}} [\varvec{\tilde{\psi }}] (\varvec{v}) + \varvec{\mathcal {K}}^\dagger [\varvec{\tilde{\psi }}] (\varvec{v}) {\varvec{\chi }}_0, b^{(k)}_j := -\sqrt{2} \tilde{m}^{(k)}_j, \, k,j=1,2,$ and $\varvec{P}_{\varvec{\vartheta }}$ denotes the orthogonal projection in direction $\varvec{\vartheta }$. The upper index on the data counts for the different incident polarizations.

### Proof

We consider $(\varvec{q},j)\in \{(\varvec{q}^{(1)},1), \, (\varvec{q}^{(1)} ,2), \, (\varvec{q}^{(2)},1), \, (\varvec{q}^{(2)},2)\}.$ Then, the system of Eq. (27) is equivalent to the four equations

34 $$\begin{aligned} \begin{aligned} \eta ^{(1)}_1 [\varvec{\vartheta }\times (\varvec{\vartheta }\times \varvec{Y} \varvec{p}^{(1)})]_1&= \tilde{m}^{(1)}_1,\quad&\eta ^{(1)}_2 [\varvec{\vartheta }\times (\varvec{\vartheta }\times \varvec{Y} \varvec{p}^{(1)})]_2&= \tilde{m}^{(1)}_2,\\ \eta ^{(2)}_1 [\varvec{\vartheta }\times (\varvec{\vartheta }\times \varvec{Y} \varvec{p}^{(2)})]_1&= \tilde{m}^{(2)}_1,\quad&\eta ^{(2)}_2 [\varvec{\vartheta }\times (\varvec{\vartheta }\times \varvec{Y} \varvec{p}^{(2)})]_2&= \tilde{m}^{(2)}_2. \end{aligned} \end{aligned}$$

Indeed, for arbitrary polarization $\varvec{q} = c_1 \varvec{q}^{(1)}+c_2 \varvec{q}^{(2)}, c_1, c_2 \in \mathbb {R}$ the expression $\eta _j [\varvec{\vartheta }\times (\varvec{\vartheta }\times \varvec{Y} \varvec{p})]_j$ can be written as a linear combination of the four expressions $\tilde{m}^{(k)}_j, k,j=1,2$:

$$\begin{aligned} \eta _1 [\varvec{\vartheta }\times (\varvec{\vartheta }\times \varvec{Y} \varvec{p} )]_1&= [c_1 \varvec{\eta }^{(1)}+c_2 \varvec{\eta }^{(2)}]_1 [\varvec{\vartheta }\times (\varvec{\vartheta }\times \varvec{Y} (c_1 \varvec{p}^{(1)}+c_2 \varvec{p}^{(2)}) )]_1\\&= c_1^2 \eta ^{(1)}_1 [\varvec{\vartheta }\times (\varvec{\vartheta }\times \varvec{Y} \varvec{p}^{(1)} )]_1 + c_1 c_2 \eta ^{(1)}_1 [\varvec{\vartheta }\times (\varvec{\vartheta }\times \varvec{Y} \varvec{p}^{(2)} )]_1 \\&\quad +\, c_1 c_2 \eta ^{(2)}_1 [\varvec{\vartheta }\times (\varvec{\vartheta }\times \varvec{Y} \varvec{p}^{(1)} )]_1 +c_2^2 \eta ^{(2)}_1 [\varvec{\vartheta }\times (\varvec{\vartheta }\times \varvec{Y} \varvec{p}^{(2)} )]_1 \\&= c_1^2 \eta ^{(1)}_1 [\varvec{\vartheta }\times (\varvec{\vartheta }\times \varvec{Y} \varvec{p}^{(1)} )]_1 + c_1 c_2 \eta ^{(2)}_1 [\varvec{\vartheta }\times (\varvec{\vartheta }\times \varvec{Y} \varvec{p}^{(2)} )]_1 \\&\quad +\, c_1 c_2 \eta ^{(1)}_1 [\varvec{\vartheta }\times (\varvec{\vartheta }\times \varvec{Y} \varvec{p}^{(1)} )]_1 +c_2^2 \eta ^{(2)}_1 [\varvec{\vartheta }\times (\varvec{\vartheta }\times \varvec{Y} \varvec{p}^{(2)} )]_1 \\&= (c_1^2 + c_1 c_2) \tilde{m}^{(1)}_1 + (c_2^2 + c_1 c_2) \tilde{m}^{(2)}_1, \end{aligned}$$

and similarly

$$\begin{aligned} \eta _2 [\varvec{\vartheta }\times (\varvec{\vartheta }\times \varvec{Y} \varvec{p} )]_2 = (c_1^2 - c_1 c_2) \tilde{m}^{(1)}_2 + (c_2^2 - c_1 c_2) \tilde{m}^{(2)}_2. \end{aligned}$$

Decomposing $\varvec{Y} \varvec{p}=\left<\varvec{\vartheta },\varvec{Y} \varvec{p}\right>\varvec{\vartheta }+\varvec{P}_{\varvec{\vartheta }} \varvec{Y}\varvec{p}$, where $\varvec{P}_{\varvec{\vartheta }}\in \mathbb {R}^{3\times 3}$ denotes the orthogonal projection in direction $\vartheta$, and using that

$$\begin{aligned} \varvec{\vartheta }\times (\varvec{\vartheta }\times \varvec{Y} \varvec{p}) = \varvec{\vartheta }\times (\varvec{\vartheta }\times \varvec{P}_{\varvec{\vartheta }} \varvec{Y} \varvec{p}) = \left<\varvec{\vartheta },\varvec{P}_{\varvec{\vartheta }} \varvec{Y} \varvec{p}\right>\varvec{\vartheta }-\varvec{P}_{\varvec{\vartheta }} \varvec{Y} \varvec{p} = -\varvec{P}_{\varvec{\vartheta }} \varvec{Y} \varvec{p}, \end{aligned}$$

the system of Eq. (34) considering (32) can be written in the form (33). $\square$

We see that Proposition 3, for $\varvec{Y} (\varvec{v})= \varvec{\tilde{\psi }}(\varvec{v}) + {\varvec{\chi }}_0 \varvec{\mathcal {K}} [\varvec{\tilde{\psi }}] (\varvec{v}) + \varvec{\mathcal {K}}^\dagger [\varvec{\tilde{\psi }}] (\varvec{v}) {\varvec{\chi }}_0$, where $\varvec{v}= \tfrac{\omega }{c} (\varvec{\vartheta }+\varvec{e}_3), {\varvec{\vartheta }}\in \mathbb {S}^2_+$, shows that the data $\tilde{m}^{(k)}_j (\varvec{v})$ for $k,j=1,2$ and two different polarization vectors $\varvec{q} = \varvec{e}_1$ and $\varvec{q} =\varvec{e}_2$ uniquely determine the projections $[\varvec{P}_{\varvec{\vartheta }} \varvec{Y} \varvec{p}^{(k)}]_j$ for $k,j \in \{1,2\}.$

Moreover, measurements for additional polarizations $\varvec{q}$ do not provide any further informations so that at every detector point, corresponding to a direction $\varvec{\vartheta }\in \mathbb {S}^2_+$, only the four elements $[\varvec{P}_{\varvec{\vartheta }} \varvec{Y} \varvec{p}^{(k)}]_j, k,j=1,2$, of the projection influence the measurements.

### Remark 2

In contrast to standard OCT where three polarization vectors were needed, see Elbau et al. (2015, Proposition 11), and to first order Born-approximation where $\varvec{Y} = \varvec{\tilde{\psi }},$ as we are going to see in the following, the above measurements due to the special form of $\varvec{Y}$ allow for reconstructing all the unknowns functions $\psi _{ij}.$

### Proposition 4

Let ${\varvec{\vartheta }}\in \mathbb {S}^2_*:=\{{\varvec{\mu }}\in \mathbb {S}^2\mid \mu _1 \ne \mu _2, \, \mu _3>0\}.$ For two given incident polarisation vectors $\varvec{q}^{(1)}$ and $\varvec{q}^{(2)},$ the system of equations (33) is equivalent to a Fredholm type system of integral equations

35 $$\begin{aligned} (\mathbb {1}+ \varvec{\mathcal {C}}) \begin{pmatrix} \tilde{\psi }_{11} \\ \tilde{\psi }_{12} \\ \tilde{\psi }_{22} \end{pmatrix}&= \varvec{b}, \end{aligned}$$

for some compact operator $\varvec{\mathcal {C}}\,{:}\,(L^2 (\varOmega ))^3 \rightarrow (L^2 (\mathbb {S}^2))^3$ and known right hand side $\varvec{b}$ depending on the OCT data. Given the solution of (35), the component $\tilde{\psi }_{33}$ satisfies a Fredholm integral equation of the first kind

36 $$\begin{aligned} \mathcal {C} \tilde{\psi }_{33} = b, \end{aligned}$$

where $\mathcal {C}\,{:}\,L^2 (\varOmega ) \rightarrow L^2 (\mathbb {S}^2)$ is a compact operator and *b* depends on the solution of (35).

### Proof

In order to reformulate equations (33), first we consider an arbitrary vector $\varvec{p}$ and we split the expression $\varvec{P}_{\varvec{\vartheta }} \varvec{Y} \varvec{p}$ into the sum

37 $$\begin{aligned} \varvec{P}_{\varvec{\vartheta }} \varvec{Y} \varvec{p} = (\mathbb {1}- \varvec{\vartheta }\varvec{\vartheta }^\top ) \varvec{\tilde{\psi }}\varvec{p} + (\mathbb {1}- \varvec{\vartheta }\varvec{\vartheta }^\top ) {\varvec{\chi }}_0 \varvec{\mathcal {K}} [\varvec{\tilde{\psi }}] \varvec{p} + (\mathbb {1}- \varvec{\vartheta }\varvec{\vartheta }^\top ) \varvec{\mathcal {K}}^\dagger [\varvec{\tilde{\psi }}] \, {\varvec{\chi }}_0 \varvec{p}, \end{aligned}$$

omitting for simplicity the $\varvec{v}$ dependence of the unknown $\varvec{\tilde{\psi }}.$

The first term on the right hand side admits the decomposition

$$\begin{aligned} (\mathbb {1}- \varvec{\vartheta }\varvec{\vartheta }^\top ) \varvec{\tilde{\psi }}\varvec{p} = \begin{pmatrix} p_1 (1 - \vartheta _1^2) &{}\quad - p_1 \vartheta _1 \vartheta _2 + p_2 (1 - \vartheta _1^2) &{}\quad -p_2 \vartheta _1 \vartheta _2 \\ - p_1 \vartheta _1 \vartheta _2 &{}\quad - p_2 \vartheta _1 \vartheta _2 + p_1 (1 - \vartheta _2^2) &{}\quad p_2 (1 - \vartheta _2^2) \\ - p_1 \vartheta _1 \vartheta _3 &{}\quad - p_1 \vartheta _2 \vartheta _3 - p_2 \vartheta _1 \vartheta _3 &{}\quad - p_2 \vartheta _2 \vartheta _3 \end{pmatrix} \begin{pmatrix} \tilde{\psi }_{11} \\ \tilde{\psi }_{12} \\ \tilde{\psi }_{22} \end{pmatrix}, \end{aligned}$$

where we observe the independence on $\tilde{\psi }_{33}.$ To analyse the other two terms, we consider (29) and define the operators acting now on the components of the matrix-valued function $\varvec{f}$:

$$\begin{aligned} \mathcal {K}_{kj} [f ] (\varvec{v})\,{:=}\, \int _{\mathbb {R}^3} \varvec{[}K^{\varvec{z}}]_{kj} (\varvec{v}; \varvec{k}) f (\varvec{k}) {\,\mathrm d}\varvec{k}, \quad \mathcal {K}_{kj}^\dagger [f ] (\varvec{v})\,{:=}\,\int _{\mathbb {R}^3} [K^{\varvec{y}}]_{kj} (\varvec{v}; \varvec{k}) f (\varvec{k}) {\,\mathrm d}\varvec{k}, \end{aligned}$$

for $k,j=1,2,3.$ Since we are interested only in the first two components of $\varvec{P}_{\varvec{\vartheta }} \varvec{Y} \varvec{p}$ and the calculations are rather lengthy we are going to omit the third component in the following expressions. The second term on the right hand side of (37) reads

$$\begin{aligned} (\mathbb {1}- \varvec{\vartheta }\varvec{\vartheta }^\top ) {\varvec{\chi }}_0 \varvec{\mathcal {K}} [\varvec{\tilde{\psi }}] \varvec{p} = \chi _0 \begin{pmatrix} p_1 \mathcal {L}_{11} &{}\quad p_1 \mathcal {L}_{12} + p_2 \mathcal {L}_{11} &{}\quad p_2 \mathcal {L}_{12} \\ p_1 \mathcal {L}_{21} &{}\quad p_1 \mathcal {L}_{22} + p_2 \mathcal {L}_{21} &{}\quad p_2 \mathcal {L}_{22} \\ *&{}\quad *&{}\quad *\end{pmatrix} \begin{pmatrix} \tilde{\psi }_{11} \\ \tilde{\psi }_{12} \\ \tilde{\psi }_{22} \end{pmatrix}, \end{aligned}$$

where

$$\begin{aligned} \mathcal {L}_{kj}\,{:=}\,(1 -\vartheta _k^2 - \vartheta _1 \vartheta _2) (\mathcal K_{1j} + \mathcal K_{2j}) - \vartheta _k \vartheta _3 \mathcal K_{3j}, \quad k,j=1,2. \end{aligned}$$

The only term where $\tilde{\psi }_{33}$ appears is the last one (as expected), namely

$$\begin{aligned}&(\mathbb {1}- \varvec{\vartheta }\varvec{\vartheta }^\top ) \varvec{\mathcal {K}}^\dagger [\varvec{\tilde{\psi }}] \, {\varvec{\chi }}_0 \varvec{p} = \chi _0 (p_1 + p_2) \\&\quad \times \begin{pmatrix} (1- \vartheta _1^2) \mathcal {M}_1 &{}\quad - \vartheta _1 \vartheta _2 \mathcal {M}_1 + (1- \vartheta _1^2) \mathcal {M}_2 &{}\quad - \vartheta _1 \vartheta _2 \mathcal {M}_2 &{}\quad - \vartheta _1 \vartheta _3 \mathcal {M}_3\\ - \vartheta _1 \vartheta _2 \mathcal {M}_1 &{}\quad (1- \vartheta _2^2) \mathcal {M}_1 - \vartheta _1 \vartheta _2 \mathcal {M}_2 &{}\quad (1- \vartheta _2^2) \mathcal {M}_2 &{}\quad - \vartheta _2 \vartheta _3 \mathcal {M}_3 \\ *&{}\quad *&{}\quad *&{}\quad *\end{pmatrix}\\&\quad \times \begin{pmatrix} \tilde{\psi }_{11} \\ \tilde{\psi }_{12} \\ \tilde{\psi }_{22} \\ \tilde{\psi }_{33} \end{pmatrix}, \end{aligned}$$

where

$$\begin{aligned} \mathcal {M}_j\,{:=}\, \mathcal {K}_{j1}^\dagger + \mathcal {K}_{j2}^\dagger , \quad j=1,2,3. \end{aligned}$$

We can combine now all the above formulas to obtain

$$\begin{aligned} \varvec{P}_{\varvec{\vartheta }} \varvec{Y} \varvec{p} = \left( \varvec{\mathcal {I}} (\varvec{p}) + \chi _0 \varvec{\mathcal {L}} (\varvec{p}) + \chi _0 (p_1 + p_2 ) \varvec{\mathcal {M}} \right) \varvec{y}, \end{aligned}$$

where

38 $$\begin{aligned} \begin{aligned} \varvec{\mathcal {I}} (\varvec{p})&= \begin{pmatrix} p_1 (1 - \vartheta _1^2) &{}\quad - p_1 \vartheta _1 \vartheta _2 + p_2 (1 - \vartheta _1^2) &{}\quad -p_2 \vartheta _1 \vartheta _2 &{}\quad 0\\ - p_1 \vartheta _1 \vartheta _2 &{}\quad - p_2 \vartheta _1 \vartheta _2 + p_1 (1 - \vartheta _2^2) &{}\quad p_2 (1 - \vartheta _2^2) &{}\quad 0 \\ *&{}\quad *&{}\quad *&{}\quad *\end{pmatrix},\\ \varvec{\mathcal {L}} (\varvec{p})&= \begin{pmatrix} p_1 \mathcal {L}_{11} &{}\quad p_1 \mathcal {L}_{12} + p_2 \mathcal {L}_{11} &{}\quad p_2 \mathcal {L}_{12} &{}\quad 0 \\ p_1 \mathcal {L}_{21} &{}\quad p_1 \mathcal {L}_{22} + p_2 \mathcal {L}_{21} &{}\quad p_2 \mathcal {L}_{22} &{}\quad 0\\ *&{}\quad *&{}\quad *&{}\quad *\end{pmatrix},\\ \varvec{\mathcal {M}}&= \begin{pmatrix} (1- \vartheta _1^2) \mathcal {M}_1 &{}\quad - \vartheta _1 \vartheta _2 \mathcal {M}_1 + (1- \vartheta _1^2) \mathcal {M}_2 &{}\quad - \vartheta _1 \vartheta _2 \mathcal {M}_2 &{}\quad - \vartheta _1 \vartheta _3 \mathcal {M}_3\\ - \vartheta _1 \vartheta _2 \mathcal {M}_1 &{}\quad (1- \vartheta _2^2) \mathcal {M}_1 - \vartheta _1 \vartheta _2 \mathcal {M}_2 &{}\quad (1- \vartheta _2^2) \mathcal {M}_2 &{}\quad - \vartheta _2 \vartheta _3 \mathcal {M}_3 \\ *&{}\quad *&{}\quad *&{}\quad *\end{pmatrix}, \end{aligned} \end{aligned}$$

and $$ \varvec{y} = \begin{pmatrix} \tilde{\psi }_{11}, \, \tilde{\psi }_{12}, \, \tilde{\psi }_{22}, \, \tilde{\psi }_{33} \end{pmatrix}^\top .$$

Then, the system of Eq. (33), considering (32) reads

39a $$\begin{aligned}{}[( \varvec{\mathcal {I}} (\varvec{p}^{(1)}) + \chi _0 \varvec{\mathcal {L}} (\varvec{p}^{(1)}) + \chi _0 \varvec{\mathcal {M}} ) \varvec{y}]_1&= b^{(1)}_1, \end{aligned}$$

39b $$\begin{aligned} _2&= b^{(1)}_2, \end{aligned}$$

39c $$\begin{aligned} _1&= b^{(2)}_1, \end{aligned}$$

39d $$\begin{aligned} _2&= -b^{(2)}_2. \end{aligned}$$

We observe that in all equations the coefficient in front of the operator $\varvec{\mathcal {M}}$ is the same, which is the only operator applying on the fourth component of $\varvec{y}.$ In addition, from (38), we see that $\vartheta _2 \varvec{\mathcal {M}}_{14} = \vartheta _1 \varvec{\mathcal {M}}_{24}.$ Thus, in order to eliminate $y_4$ we reformulate the above system as follows: we subtract from Eq. (39a) the Eq. (39c), from Eq. (39b) the Eq. (39d) and from $\vartheta _2 \cdot$ (39a) the equation $\vartheta _1 \cdot$ (39b), resulting to

$$\begin{aligned}{}[( \varvec{\mathcal {I}} (\varvec{p}^{(1)} -\varvec{p}^{(2)} ) + \chi _0 \varvec{\mathcal {L}} (\varvec{p}^{(1)}-\varvec{p}^{(2)}) ) \varvec{y}]_1&= b^{(1)}_1 - b^{(2)}_1, \\ [( \varvec{\mathcal {I}} (\varvec{p}^{(1)} -\varvec{p}^{(2)} )+ \chi _0 \varvec{\mathcal {L}} (\varvec{p}^{(1)}-\varvec{p}^{(2)}) ) \varvec{y}]_2&= b^{(1)}_2 + b^{(2)}_2, \\ \vartheta _2 [( \varvec{\mathcal {I}} (\varvec{p}^{(1)}) + \chi _0 \varvec{\mathcal {L}} (\varvec{p}^{(1)}) + \chi _0 \varvec{\mathcal {M}} ) \varvec{y}]_1 \\ - \vartheta _1 [( \varvec{\mathcal {I}} (\varvec{p}^{(1)}) + \chi _0 \varvec{\mathcal {L}} (\varvec{p}^{(1)}) + \chi _0 \varvec{\mathcal {M}} ) \varvec{y}]_2&= \vartheta _2 b^{(1)}_1 - \vartheta _1 b^{(1)}_2. \end{aligned}$$

The above system in compact form reads

40 $$\begin{aligned} (\varvec{\tilde{\mathcal {I}}} + \varvec{\mathcal {N}} ) \varvec{\tilde{y}} = \varvec{\tilde{b}}, \end{aligned}$$

where

$$\begin{aligned} \begin{aligned} \varvec{\tilde{\mathcal {I}}}&= \frac{\mathrm{i}}{2} \begin{pmatrix} 2(\vartheta _1^2 -1) &{}\quad 2 (1+ \vartheta _1 \vartheta _2 -\vartheta _1^2 ) &{}\quad -2\vartheta _1 \vartheta _2 \\ 2\vartheta _1 \vartheta _2 &{}\quad 2(\vartheta _2^2 -\vartheta _1 \vartheta _2 - 1 ) &{}\quad 2(1-\vartheta _2^2 ) \\ -\vartheta _2 (1+\mathrm{i}) &{}\quad \vartheta _1 (\mathrm{i}+1) + \vartheta _2 (1-\mathrm{i}) &{}\quad -\vartheta _1 (1-\mathrm{i}) \end{pmatrix},\\ \varvec{\mathcal {N}}&= \mathrm{i}\chi _0 \begin{pmatrix} -\mathcal {L}_{11} &{}\quad \mathcal {L}_{11} - \mathcal {L}_{12} &{}\quad \mathcal {L}_{12} \\ -\mathcal {L}_{21} &{}\quad \mathcal {L}_{21} - \mathcal {L}_{22} &{}\quad \mathcal {L}_{22} \\ \phantom {-}\mathcal {N}_1 &{} \quad \mathcal {N}_2 &{} \quad \mathcal {N}_3 \end{pmatrix}, \\ \varvec{\tilde{y}}&= \begin{pmatrix} y_1 \\ y_2 \\ y_3 \end{pmatrix}, \quad \varvec{\tilde{b}} = \begin{pmatrix} b^{(1)}_1 - b^{(2)}_1 \\ b^{(1)}_2 + b^{(2)}_2 \\ \vartheta _2 b^{(1)}_1 - \vartheta _1 b^{(1)}_2 \end{pmatrix}, \end{aligned} \end{aligned}$$

and

$$\begin{aligned} \mathcal {N}_1&:= \tfrac{1}{2} [(1+\mathrm{i}) (\vartheta _1 \vartheta _2\mathcal {L}_{21} -\vartheta _2^2 \mathcal {L}_{11} )-2\mathrm{i}\vartheta _2 \mathcal {M}_1 ],\\ \mathcal {N}_2&:= \tfrac{1}{2} [(1-\mathrm{i})(\vartheta ^2_2 \mathcal {L}_{11} - \vartheta _1 \vartheta _2 \mathcal {L}_{21}) - (1+\mathrm{i}) (\vartheta ^2_2 \mathcal {L}_{12} - \vartheta _1 \vartheta _2 \mathcal {L}_{22}) \\&\phantom {:=}\quad -2\mathrm{i}\vartheta _2 \mathcal {M}_2 + 2\mathrm{i}\vartheta _1 \mathcal {M}_1 ],\\ \mathcal {N}_3&:= \tfrac{1}{2} [(1-\mathrm{i}) (\vartheta _2^2 \mathcal {L}_{12} -\vartheta _1 \vartheta _2 \mathcal {L}_{22}) +2\mathrm{i}\vartheta _1 \mathcal {M}_2 ]. \end{aligned}$$

We compute the determinant of $\varvec{\tilde{\mathcal {I}}}$ which is given by

$$\begin{aligned} \det (\varvec{\tilde{\mathcal {I}}})&= -\tfrac{\mathrm{i}}{8} \left( -\vartheta _1^3 + \vartheta _1^2 \vartheta _2 - \vartheta _1 \vartheta _2^2 +\vartheta _1 + \vartheta _2^3 - \vartheta _2 \right) \\&= -\tfrac{\mathrm{i}}{8} (\vartheta _2 - \vartheta _1) (\vartheta _1^2 + \vartheta _2^2 -1). \end{aligned}$$

Recall that $\varvec{\vartheta }\in \mathbb {S}^2_+,$ meaning $\vartheta _3 >0.$ Then, if in addition we impose that $\vartheta _1 \ne \vartheta _2$ for all $\varvec{\vartheta }\in \mathbb {S}^2_+,$ the matrix $\varvec{\tilde{\mathcal {I}}}$ is invertible with $\varvec{\tilde{\mathcal {I}}}^{-1} = \det (\varvec{\tilde{\mathcal {I}}})^{-1} \text{ adj } (\varvec{\tilde{\mathcal {I}}}).$ Then, Eq. (40) can be written in the form

41 $$\begin{aligned} (\mathbb {1}+ \varvec{\tilde{\mathcal {I}}}^{-1}\varvec{\mathcal {N}} ) \varvec{\tilde{y}} = \varvec{\tilde{\mathcal {I}}}^{-1}\varvec{\tilde{b}}, \end{aligned}$$

which is the Fredholm integral equation of the second kind (35), for $\varvec{\mathcal {C}}\,{:=}\,\varvec{\tilde{\mathcal {I}}}^{-1}\varvec{\mathcal {N}} ,$ and $\varvec{b}\,{:=}\,\varvec{\tilde{\mathcal {I}}}^{-1}\varvec{\tilde{b}}.$ Once (41) is solved for $y_1,y_2$ and $y_3$ we can choose one of the four equations from the system (39) resulting to a Fredholm integral equation of the first kind for the unknown $y_4$ now:

$$\begin{aligned} \mathcal {M}_3 y_4 = b, \end{aligned}$$

for some known function *b*,  depending on $\varvec{\tilde{y}}$ and $\varvec{\tilde{b}}.$ This is Eq. (36) for $\mathcal {C} := \mathcal {M}_3.$

The compactness of the integral operators $\varvec{\mathcal {C}}$ and $\mathcal {C}$ follows from the compactness of the operators $\varvec{\mathcal {K}}$ and $\varvec{\mathcal {K}}^\dagger ,$ see Lemma 2, since they are operators that act on the components of the matrix-valued function. $\square$

### Remark 3

Equation (36) reflects the ill-posedness of the inverse problem, due to the compactness of the integral operator.

## Conclusions

In this work we have formulated the inverse problem of recovering the electric susceptibility of a non-magnetic, inhomogeneous orthotropic medium, placed in a polarized-sensitive optical coherence tomograph (PS-OCT) as a system of Fredholm integral equations (both of first and second kind). Under the assumptions of a non-dispersive, weakly scattering medium with small background variations we have shown that we can reconstruct all the coefficients of the matrix-valued susceptibility, given the data for two different incident polarization vectors. This paper can be seen, on one hand, as a first attempt to model mathematically PS-OCT and on the other hand, as a theoretical basis for an upcoming paper where the numerical validation of the proposed method will be examined.
